# Development of pre-service early childhood teachers’ technology integrations skills through a praxeological approach

**DOI:** 10.1186/s41239-022-00344-8

**Published:** 2022-07-28

**Authors:** Taibe Kulaksız, Mehmet Toran

**Affiliations:** 1 Distance Education Application and Research Center, University of Health Sciences, Hamidiye Campus, Selimiye Neighborhood, Tıbbiye Street, Nu:38, Üsküdar, 34668 Istanbul, Turkey; 2 Early Childhood Education Department, Istanbul Kültür University, Istanbul, Turkey

**Keywords:** Instructional technology, Pre-service teachers, Technology integration, Early childhood education, Praxeological research

## Abstract

How to improve and what should be carried out for pre-service teachers’ technological competencies for teaching purposes is still an important issue on the agenda of the higher education field. In light of this, we aimed to reflect the individual and collective technology integration knowledge and skills construction process of pre-service early childhood education teachers with democratic participation. We utilized the praxeological approach as a method and learning approach to reveal the reflections of the instructional technologies course. The participants in this study were 52 sophomore pre-service teachers in the early childhood education department. We collected the data from various sources such as interviews, portfolios, researchers’ field notes, e-mails, online course evaluation form. We carried out the thematic analysis method to analyze the data. The findings indicated that three main themes emerged as initial challenges, learning process, and learning outcomes during enhancement of pre-service early childhood teachers' technology integration knowledge and skills. As a result, the praxeological approach used in instructional technologies courses in teacher education programs leads to a crucial digital transformation to be ready to become future teachers.

**Supplementary Information:**

The online version contains supplementary material available at 10.1186/s41239-022-00344-8.

## Introduction

The use of technology in education has been getting increased. Although the use of technology in early childhood education (ECE) is controversial, it is helpful for children learning and development when it is used to create natural learning environments to meet their needs and for teaching purposes (Bolstad, 2004; Van Scoter et al., 2001). Accordingly, research on the effectiveness of technology in ECE has been produced positive results (Kerckaert et al., 2015). The various instructional technologies such as digital storybooks, and digital games, improve children's technical, literacy, social, math, problem-solving, and emotional skills (Bolstad, 2004; Contini et al., 2018; Morfoniou et al., 2020; Verbruggen et al., 2021; Zomer, 2014), therefore ECE teachers should mediate digital technologies wisely for children’s understandings (Segal-Drori & Ben Shabat, 2021). Technology also allows collaboration between children and children-adults (Bolstad, 2004). Furthermore, it is observed that digital competencies (e.g. digital citizenship) have been also included in preschool education programs (Lauricella et al., 2020). Being prepared for future learning and teaching scenarios is essential beyond focusing only on the present conditions of schools (Dong & Xu, 2020; Seufert et al., 2021). Therefore, technology knowledge and skills for educational purposes seem more important than all times for future ECE teachers.

On the other hand, teachers have low-level knowledge and skills about how to use technology in ECE (Contini et al., 2018; Martín et al., 2020; Masoumi, 2021; Öner, 2020). Moreover, studies reveal that some of them have negative attitudes towards technology use in ECE (Dong & Xu, 2020; Öner, 2020). However, Zilka's (2021) study indicated that pre-service teachers have positive attitudes, and they are more likely to integrate technology in ECE when they begin working more than in-service teachers. Therefore, technology integration in education of teacher candidates has required a systemic change in several ways discussed by Tondeur et al. (2012) such as technology planning and leadership, role models, collaboration, feedback, instructional design, authentic experiences. To complement this need, the praxeological approach, which refers to acquirement useful knowledge and skill as a transformation process (Pascal & Bertram, 2012), were taken into account to design an instructional technologies course for pre-service ECE teachers.

## Theoretical background

### Developing pre-service teachers’ technology integration knowledge and skills

The studies show that pre-service teachers' knowledge and skills on technology integration increase with courses included in teacher education programs (Jung & Ottenbreit‐Leftwich, 2020; Neumann et al., 2021; Schina et al., 2020). Therefore, training seems like a key element to enhance pre-service teachers’ digital competence for education (Romero-Tena et al., 2020). However, ECE teacher education programs in terms of technology integration are still challenging, even if pre-service teachers have courses, they do not feel enough competent to transfer their knowledge and skills to the next implementations (Masoumi, 2021).

Besides, there are more factors linked to the construction process of technological knowledge and skills. Pre-service teachers' ability to integrate technology into ECE is closely related to their attitudes and their perception of the process (Zilka, 2021). Pre-service ECE teachers having low positive attitudes may not be aware of the role of technology in ECE, as their attitudes towards technology are related to information and communication technologies (ICT) usage, ICT professional training, and ICT skills (Dong & Xu, 2020). As the development of pre-service teachers' technology skills is affected by different factors as mentioned, these education programs for pre-service ECE teachers are more beyond merely acquiring digital skills (Masoumi, 2021). Hence, the non-negligible factors influencing their technology integration skills and knowledge should be also included in the pre-service teachers’ education programs.

Some approaches and strategies are suggested for pre-service teachers’ education in the context of technology. Tondeur et al. (2012) proposed a model called Synthesize Qualitative Data Model based on an intensive literature review. The model suggests the important elements for pre-service teachers’ preparation for technology in education like aligning theory and practice, teacher educators as role models, reflecting on attitudes about the role of technology in education, learning technology by design, collaborating with peers, scaffolding authentic technology experiences, moving from traditional assessment to continuous feedback. Polly and Byker (2020) also suggested collaborative experiences, appropriate scaffolds, and focused learning goals for pre-service teachers’ technological pedagogical content knowledge improvement by using the zone of proximal development. Providing quality examples, gaining experience in class, and feeling motivated and supported during design process strategies are valuable to improve the digital skills of pre-service teachers, however, the inclusion of these strategies in teacher education seems like a complex process (Howard et al., 2021).

However, the problems encountered from teacher education programs were reported as (Masoumi, 2021): (1) not engaging in activities unless they are compulsory, (2) not being aware of the importance of technology experience opportunities during the lessons, (3) not feeling comfortable using technology in education even after training, (4) the limitations of teacher educators about providing good examples and being a role model. So that, teacher education programs should concentrate on enhancing their perceptions of technology usability and providing context-specific examples and tools to develop technology-related skills to support of changing environments of the children (Dong & Xu, 2020; Xie et al., 2019). As a result, the development of technology integration for pre-service teachers is a dynamic and complex process, which shows the praxis side of the field. It involves not only focusing on knowledge and skills but also psychosocial change for them. Hence, in the light of the literature, instructional technologies course for pre-service ECE teachers was designed and developed holistically, with a praxeological approach as a social transformation process by considering context.

### Praxeological approach

The views of research practice are required to change with a more participatory lens to address the ongoing challenges, troubles, and insufficiency that we face in studies, although practitioners' theory and practice and practice-based research have been widely accepted (Pascal & Bertram, 2012). In addition, the domination of the evidence-based paradigm research reveals the pre-determined outcomes of the studies, therefore, the process might be mainly undemocratic due to reliance on researcher(s) hands (Vandenbroeck et al., 2012). However, the praxeological approach overwhelms some limitations by picturing authentic complicated circumstances, in other words, the reality.

Praxeological perspective represents the mixture of action (praxis), reflection (phronesis), power (politics), and values (ethics) (Pascal & Bertram, 2012). According to Pascal and Bertram (2012), praxeological research has two purposes. The first purpose is to produce beneficial knowledge and skills, which are situated in a specific context with a participatory and democratic sense. The second purpose is to encourage the transformation process of building self-awareness and self-critique through an individualized path of the person. The strengths of this approach can be listed as follows (Pascal & Bertram, 2012); participants can define the way to advance themselves, take responsibility for their actions, stimulate collaborative learning, respond to questions regarding implementation, have transparent ethics and values. Another benefit of the praxeological approach provides an opportunity to advocate change and transformation by taking into account the identification of their context and pedagogical practice (Winterbottom & Mazzocco, 2016).

As a research method, praxeology occurs as an alternative method in educational studies to monitor change and construction of knowledge in a real environment (Formosinho & Oliveira-Formosinho, 2012). Also, the researcher(s) employing the praxeological method can reflect the complexity of the real context by setting various methods on revealing the participants' stories (Pascal & Bertram, 2012). Additionally, as an instructional strategy, praxeological learning provides a potential theoretical framework for teacher education programs to develop and support pre-service teachers’ pedagogical knowledge and skills by taking part in authentic experiences (Winterbottom & Mazzocco, 2016). Thus, the learning process allows students to overcome challenges collaboratively among their community, understand the diversity, voice their ideas (contrary instructor’s less), follow the process by assessing themselves, and put forward their particular goals (Winterbottom & Mazzocco, 2016).

The context of this study involves the elements of contemporary instructional technologies, technology integration in ECE, and pre-service ECE teachers' digital competencies. Under these current circumstances of the Covid-19 outbreak, accessibility of the technology has been maximized, the necessity of the technology integration in ECE became obvious, and taking the responsibility of own learning has come to the forefront. Therefore, neglected problems, as mentioned above by pre-service ECE teachers to enhance their competency was embodied in this study based on today’s needs. From this starting point, pre-service teachers revealed the course objectives collectively based on a prediction of the professional development on technology integration needs of today and future preschool teachers. The course was designed according to these objectives with the researchers by involving the pre-service teachers' suggestions about content, teaching methods and techniques, and assessment/evaluation approaches of the course. Thus, pre-service teachers were encouraged to experience possible professional development activities both individually and with their peers (as future colleagues). Therefore, this study is important to support technology integration in ECE teacher education and to expose a digital transformation process by eliminating the gap between theory and practice, which is frequently emphasized in teacher education studies.

### Purpose of the research

This study aims to reflect the individual and collective technology integration knowledge and skills construction process of pre-service ECE teachers in the instructional technologies course within democratic participation.

In this context, the following research questions were sought answers:

1. What are the pre-service teachers' perceptions about technology in ECE at the beginning of the instructional technologies course?
2. How is the instructional design for the instructional technologies course that the pre-service teachers put forward collectively based on their perceptions?
3. How do the pre-service teachers' perceptions about technology in ECE change during the instructional technologies course?
4. How do pre-service teachers consider praxeological learning experience in instructional technologies course at the end of the semester?

## Method

This study employed a praxeological approach which aims to acquire directly useful knowledge and skills for participants and allows them to support their social transformation in the knowledge construction process (Pascal & Bertram, 2012). Praxeology was used as a research methodology since it provides an authentic procedure of learning within a socio-cultural context because of aligning with the participatory paradigm. In this way, both participants and researchers were located as subjects of the study to uncover the real environment without pre-determined interventions. Therefore, praxeology intertwined a research method and a learning approach naturally. In other words, in this study, instructional technologies course development based on a praxeological approach manifests itself in the concept of technology integration knowledge and skills of pre-service ECE teachers or vice versa.

### Participants and context of the study

In Turkey, technology integration in ECE was not common until the need for emergency distance education emerged due to the lockdown of COVID-19. On the contrary, there has been a tendency to research and practice in this area. In line with these facts, this research was conducted in the instructional technologies course remotely. The nature of praxeological research requires participation, being democratic, and collaboration (Pascal & Bertram, 2012), therefore, both researchers and pre-service teachers are positioned as subjects of this study in allegiance with the praxeological method.

This study consists of 52 sophomore pre-service teachers (51 females, 1 male) in the early childhood education department, three of them took the course before. Their ages were between 19 and 32. The academic background of the group consisted of mandatory courses in teacher training program; which were pedagogical knowledge (e.g., introduction to early childhood education, educational psychology), pedagogical content knowledge (e.g. science education in early childhood, mathematics education in early childhood), and technological knowledge (limited with mandatory information technologies course) courses (exception, two students has graphic design background). Pre-service ECE teachers’ roles in this study -as agreed in the first week- are to follow up with the course, involving discussion and implementations, supporting peers, taking responsibility for their learning individually.

Another type of participant in this study is the researcher. While the first author is specialized in educational technology and acted as an instructor of the course, the second author is specialized in early childhood education and has been the advisor of the pre-service teachers for the past two years. As researchers, we followed Pascal and Bertram’s (2012) praxeological researcher’s guiding principles: (1) Value-driven, (2) Democratic and participatory, (3) Critical, (4) Subjective, (5) Methodologically rigorous, (6) Action-based. To do this, researchers were followed a value-driven approach that uncovers pre-service ECE teachers’ attitudes and beliefs regarding technology, advocates their voices for inclusion and collaboration, is critical and subjective for acknowledging multiple perspectives for equitability, and action-based research for creating a dynamic learning process of praxis. Therefore, the researchers encouraged the students to involve, criticize, support, create to comprehend the technology integration in ECE.

### The starting point of the course design process

**Fig. 1:**
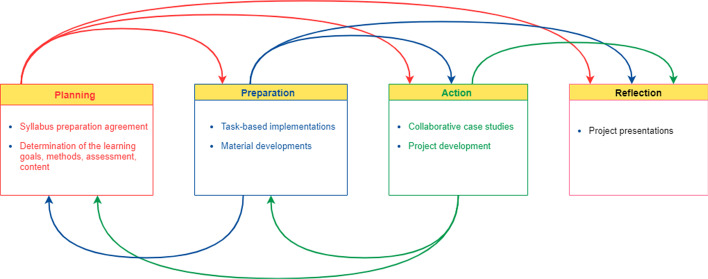
Overview of the instructional technologies course dynamics

In the beginning, the instructional technologies course including instructional elements, such as content, learning goals, methods, assessment, was unclear as we adopted the praxeological approach. This uncertainty caused an ill-structured and dynamic process. However, before the semester, the researchers exchanged ideas about the students' academic backgrounds for the prediction of their expectations, which concluded with Fig. 1 as a starting point representing the outline of the course. Therefore, researchers’ experience and collaboration formed the basis of the research.

In the first week, pre-service teachers' views on instructional technologies, their anticipations and goals about the course, their preferences in learning/teaching methods, and assessment-evaluation were asked. Also, the instructor’s and students' responsibilities were listed to achieve their goals. Encouraging democratic participation in course planning, these answers were collected anonymously with interactive presentations. The data were used to determine the goals, content, teaching method, and assessment-evaluation of the course in the following weeks shown in Additional file 1.

On the other hand, praxeological learning involving planning, preparation, action, and reflection components, is a strategy to meet learning outcomes and content (Winterbottom & Mazzocco, 2016). As seen in Fig. 1, planning, preparation, action, and reflection steps were performed consecutively, but also rotationally sometimes, which was shown by arrows, because of indetermination. Planning and preparation happened within 1–3 weeks, while preparation of the content, materials, and activities put in action during the remote lessons within 3–8 weeks. Collaborative activities and projects were mainly performed between 9 and 11 weeks. Lastly, the reflection part was included presentations and feedback. The course was carried out in two sections as A (N = 25) and B (N = 27) groups remotely.

### Data collection process

Various qualitative data collection methods were used in this study following the praxeological approach to reflect the richness and complexity of that process (Pascal & Bertram, 2012). Therefore, every possible data source was included in the study. Video recordings (synchronous lectures), interactive slides, pre-service ECE teachers’ works (portfolios and projects), e-mails, researchers’ field notes, online course evaluation form, and semi-structured interview form were used as data collection tools.

- *Synchronous lectures* were recorded as videos via a learning management system every week (N_video_ = 30).
- The first week of the semester, *online interactive slides* (Poll Everywhere) were used to collect pre-service ECE teachers’ opinions about course design anonymously.
- *Students’ works* included two main elements as portfolio and project. Firstly, the digital materials and reports individually developed by pre-service ECE teachers were compiled as a portfolio with a self-assessment report (N_portfolio_ = 52) and collected at the eight-week of the course. Secondly, the final project following the instructional design steps was developed individually or in groups of two (N_project_ = 35) and gathered at twelve-week of the course.
- *E-mails* of the pre-service ECE teachers between the lecturer/advisor of the course also followed during the semester to comprehend the off-line activities.
- The *researcher’s field notes* were composed regarding the teaching/learning process in the course every week.
- End of the semester, an *Online Course Evaluation Form* was designed via Google Forms to obtain anonymously pre-service ECE teachers' opinions and suggestions about course content, teaching method, learning environment, and also demographics (N_form_ = 37).
- A *Semi-structured Interview Form* (Additional file 2) consisting of 6 questions was designed to conduct interviews via Zoom online meeting platform about the learning experiences of the pre-service teachers 3–4 weeks after the course-end (N_interview_ = 7).

### Data analysis

Variety of the data collection tools, in other words, the multiple sources of data enabled the representations of the authentic experiences with enrichment of the context. Therefore, thematic analysis was used in this study to reveal the sense of data. This method concentrates on identifying, organizing, and representing the common meaning (theme) of the dataset in the context of research questions rather than specific meaning within single data (Braun & Clark, 2012). Braun and Clark’s (2012) six-phase approach was followed: (1) Familiarizing yourself with the data: It aims to get familiar with the dataset content which requires enough reading and re-reading until the researcher(s) absorption. (2) Generating initial codes: It defines the labelling of the content to describe the potential feature of the data regarding research questions. (3) Searching for themes: It is an evolving process by shifting the codes to the theme(s) to refine the dataset. (4) Reviewing potential themes: As an iterative process that emerged themes are reviewed to decide essential themes. (5) Defining and naming themes: The final themes were named and clarified the means of each theme by describing. (6) Producing the report: It means convincingly representing the themes to reflect the whole picture of the study.

The data analysis started with interviews by taking into account Braun and Clark's (2012) procedure. Firstly, the interviews were transcribed and read in detail line by line repeatedly. Secondly, the initial codes were created by one of the researchers. Afterwards, initial codes were re-examined by the other researcher, and final codes were decided together in order to resolve the disagreements in the last phase of the coding (Miles & Huberman, 1994). Thirdly, possible themes were emerged from the codes by using an inductive approach (Patton, 2002). Fourthly, all data were reviewed for consistency according to the possible themes for the last determination. Fifthly, the final themes were named and defined to be able to reflect the purpose and questions of the research. Sixthly, the final themes and definitions were reported with multiple data sources in the title of findings based on research questions. As a result, a good thematic analysis includes the themes which are a simple and singular focus, associated with each other without overlapping, and directly related to research questions (Braun & Clark, 2012). Therefore, our final analysis was presented by visualizing in the findings section as each theme built upon each other, which was connected to research questions.

Cresswell and Creswell’s (2018) suggested strategies about validity and reliability were followed to ensure the accuracy and credibility of the study. Various types of data were collected as evidence for the solid construction of the themes and consistency via triangulation. Thick descriptions and biases, within context and participants' background definition, were presented for the enrichment of each theme accompanied by direct quotations from different participants and data sources to delineate the findings. Furthermore, the first author of the study spent prolonged time because of conducting the course, while the second author involved the data analysis process as a peer debriefer to avoid biases of the first author. Lastly, transcription of the interviews, iterative reading, and comparison of the codes/themes during thematic analysis and final cross-checking and consensus were generated consistent findings.

The praxeological approach also guided the data collection and analysis in some aspects. The unclearness of the starting point of the research and update in progress capacity because of democratic participation yielded all possible sources to be considered as data collection tools. Then, these multiple sources contributed both to increase validity and reliability and to reflect research and learning/teaching progress in the course. Therefore, this approach allowed to be structured all the phenomena that emerged in the context of both research and educational activities with all their naturalness, and to reveal the reality at the maximum level.

### Findings

**Fig. 2:**
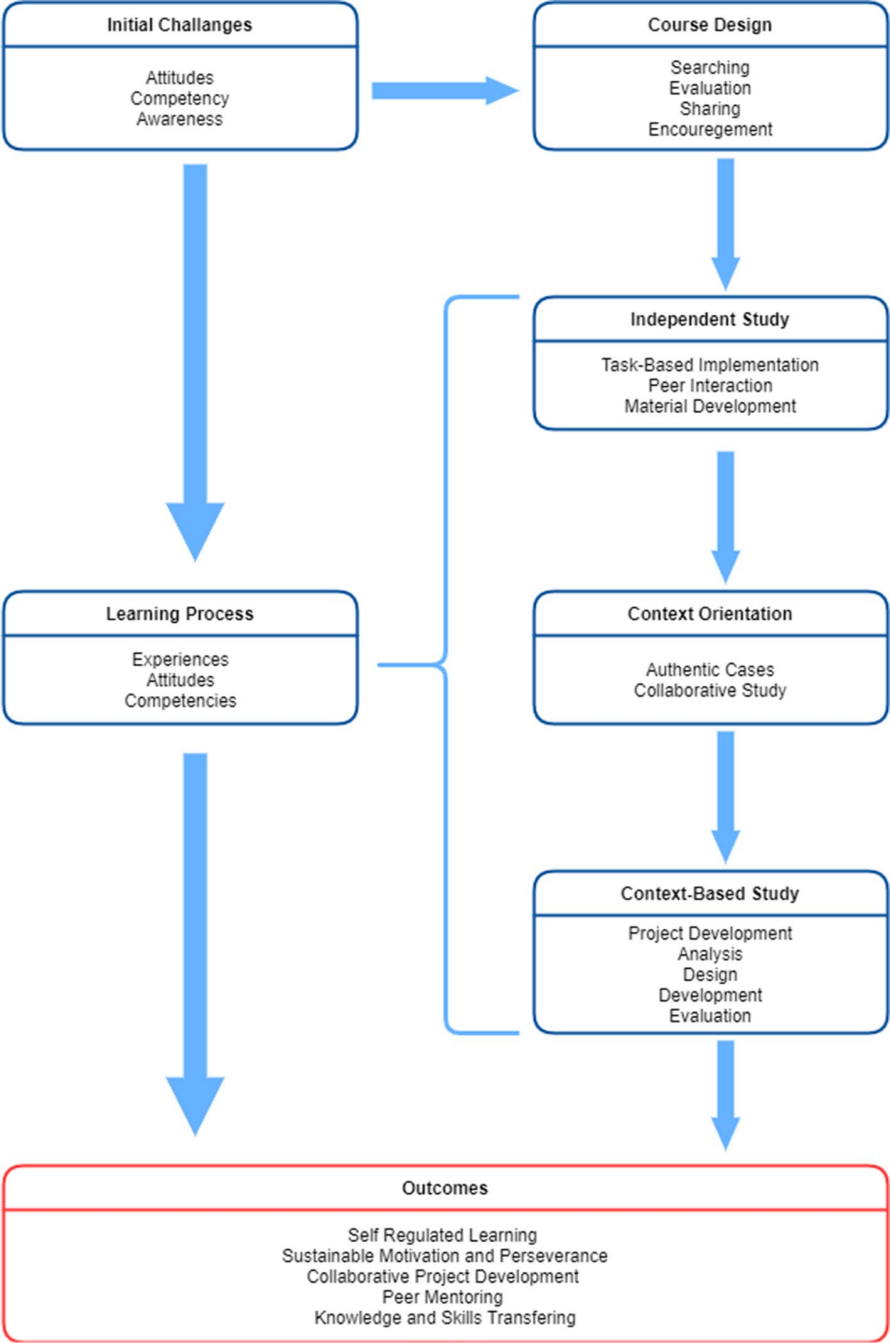
Instructional technologies course design based on praxeological approach

Pre-service ECE teachers’ technology integration knowledge and skills development process in the context of instructional technologies course through praxeological approach was examined. As a result of the analysis of the data, three themes (Fig. 2) emerged as initial challenges, learning process, and learning outcomes given in detail as follows.

### Initial challenges: what are the pre-service teachers' perceptions about technology in ECE at the beginning of the instructional technologies course?

The initial challenges that represent the pre-service teachers' perceptions about technology in ECE were attitudes, skills, and unawareness of educational technology as an answer to the first question of the research. These perceptions were revealed during a three-week course using the following activities.

The beginning of the course was partially dominated by syllabus preparation taking into account ethical issues in terms of decisions of learning goals, content, teaching/learning method, course evaluation. Therefore, a bond was aimed to be established regarding privacy and trust, so that pre-service teachers could freely express their thoughts. Sharing feelings was encouraged by the instructor, including online/offline communications. Syllabus preparation took three weeks, as pre-service teachers were not familiar with making decisions and taking responsibility for course content and teaching methods. During this time, pre-service teachers expressed their prior knowledge levels, wishes, and hesitations, depending on the strengthening of the established bond. It was observed that pre-service teachers were highly influenced by each other's ideas; they were afraid of sharing negative thoughts. The pre-service ECE teachers' suggestions for course content were mostly limited to the "technology" word due to the unfamiliarity of specific apps, which was considered as a clue for their low level of proficiency and awareness about teaching with technology. Also, it was determined that pre-service teachers had high expectations from the course, prefer experience-based teaching techniques, collaborative studies, product-oriented outputs (materials), and process-oriented assessments (projects, homework) for the course evaluation. In addition, the responsibilities of students and teachers were discussed to achieve the pre-determined goals. Anonymous answer from first-week interactive slides shared:

As a student, to participate effectively in the lesson and do the assigned tasks. As a teacher, to share your knowledge and experience with us in the most instructive way, to support problems quickly. (Anonymous, from interactive slides)

Lastly, the draft syllabus was reached with students' approvals, however, it was emphasized by the instructor that changes could be made in the scope of the syllabus in line with their demands and needs during the semester.

As a result of this phase, pre-service ECE teachers' initial challenges at the beginning of the instructional technologies course were revealed as attitudes, skills, and unawareness of educational technology. These emerged from the data collected during class discussions, anonymously answered questions presented in interactive slides, and interviews conducted. Their attitudes and competencies differed between technology and educational technology. Examining the attitudes of pre-service ECE teachers, they seemed biased and worried about technology, while they had positive attitudes and low self-efficacy regarding educational technology. P-3 mentioned her feelings at the interview:

I consider myself very inadequate about technology. Frankly, I was wondering about what to do. I was anxious ... I thought I would be unsuccessful. (P-3, from interview)

Meanwhile, pre-service teachers claimed that most of them had low technology and educational technology skills because of unfamiliarity and lack of awareness in the field. Some of them suffered even in the use of the basic functions of the devices (tablets, laptops) and the programs (Microsoft Word, PowerPoint). P-7 admitted at the interviews that:

I faced difficulties at some points due to the inability to adapt. I was not familiar with the common technologies. It was so hard for me. (P-7, from interview)

Moreover, it was revealed that pre-service teachers also had inadequacies in using technology in education, especially in the first few weeks of conducting online task-based implementations and individual material development studies. Although pre-service teachers considered themselves inadequate in the use of technology in education, it was observed frequently during in-class talks that they also had the curiosity and motivation about that. As P-1 mentioned about the necessity of the course during interviews:

We live in the technology age. We should not be left behind, and use technology effectively. But we did not know how to reach the kids over technology, indeed. (P-1, from interview)

### Learning process: how is the instructional design for the instructional technologies course that the pre-service teachers put forward collectively based on their perceptions?

The learning process, in other words, the design of the course was constructed by pre-service ECE teachers collectively based on their decisions over their perceptions as attitudes, skills, and unawareness about technology integration in education. The instructional design of the course within democratic participation fulfilled the second research question.

The learning process lasted 14 weeks, consisting of four parts (see Additional file 1). Firstly, the introductory knowledge about the educational technology field was presented by the instructor in parallel with the syllabus design. Secondly, independent studies that include online task-based learning, group interactions, offline material development activities were employed. It was aimed to develop pre-service ECE teachers' technology knowledge, and have them experience the applications during the online part of the course. While they were divided into online break-out rooms, including 4–5 people, task-based implementation lists were provided to complete the task with peers and instructor support. Although they asked for support from the instructor during break-out room studies for the first several weeks, the support request declined over time as they became able to solve the problems by themselves (researcher’s notes, sixth week). As offline, they developed digital material based on the learning outcome(s) in the ECE curriculum in Turkey that they chose. Every week, volunteer pre-service ECE teachers shared their digital materials to get feedback and discuss them publicly. Thirdly, contextual orientation studies were conducted to adopt different educational environments for technology integration. To achieve this goal, students were given different authentic case studies to determine problems, assumptions, and alternative solutions during group discussions. Fourthly, pre-service ECE teachers (individually or in a two-person group) were required to develop digital materials using new applications (other than the ones they learned during the course) based on the methods of analysis, design, development, implementation, and evaluation steps. The implementation step was done if the pre-service ECE teachers had a chance to reach at least one target audience. Last four weeks, each project was presented and given feedback by the instructor and peers. These projects aimed to reflect their technology integration skills and knowledge and the ability to learn new programs by themselves.

### Learning process: how do the pre-service teachers' perceptions about technology in ECE change during the instructional technologies course?

As a result of the learning process phase, the third research question was also answered. Pre-service teachers shed light on their learning process during the course in three components as attitudes, experience, and competencies. The instructional technologies course was designed by adopting a praxeological learning approach to allow them to be the subject of their learning journey. Therefore, it revealed that this self-designed course had a noticeable change in their attitudes, experience, and competencies regarding technology integration in ECE. It was stated by pre-service ECE teachers and also observed by instructors that the crucial experience in the course was active participation in the learning process. P-7 emphasized the importance of the relationship among the course participants at the interview that:

It was very good for us that our teacher shared this process with us, got our opinions, gave us feedback, evaluated our suggestions. It was quite good for us to have a collaborative interaction. (P-7, from interview)

At the same time, it was determined that peer mentoring and peer interaction were quite intense. Active participation, peer mentoring and interaction, and attitudes changing can be explained by the fact that pre-service ECE teachers hold decisive roles in the progress. Collaboration, sharing, helping each other, and solidarity have enabled the formation of collective learning, which amplified their learning responsibility. As voiced in the interview with P-1 is that:

The mentor [break-out room lead] was running the group. We were asking her/him, when we could not do some tasks, for example, “I couldn't do step 3 [in the task list]. How did you do?”. She/he showed us via screen-sharing how to do it. When the mentor was not able to do the task, she/he gave the screen-sharing to the one who did it. (P-1, from interview)

The collaborative studies had a positive impact on their educational technology competencies. It was reflected in their portfolios, researcher’s observations, and interviews with the participants that pre-service ECE teachers can carry out independent projects, design and develop digital materials, and make self-assessments. The initiatives taken by the pre-service teachers in the learning process have stimulated them to use educational technologies effectively and to learn by doing. The effect of the participants' accomplishments during the course was expressed as follows:

I was encouraged as I completed the project. I had been one who was unable. However, towards the end of the semester, I started helping my friends, giving ideas and feedback. Although I am incredibly prejudiced, I can say that practicing made me free from all my prejudices. (P-5, from interview)

For me, the first two weeks and after were very important. As I accomplished things, I no longer fear the next application given by our teacher. (P-3, from interview)

### Learning outcomes: how do pre-service teachers consider praxeological learning experience in instructional technologies course at the end of the semester?

The last research question answers at the end of the fourteen-week experience learning outcomes on pre-service ECE teachers were sustainable motivation and perseverance, self-regulated learning, collaborative project development, peer mentoring, and knowledge and skill transfer to different contexts. At the end of the semester, pre-service ECE teachers referred to the bond established during the semester as “warm”, and this bond helped to get these learning outcomes.

Pre-service ECE teachers’ studies, researchers’ observations, and interviews have clearly shown that their interest and attitudes regarding technology in education were increased. The findings indicated that their turning points were mostly noticed in the second or third independent studies. Therefore, it is possible to say that positive attitudes have eliminated prejudices and concerns, fostered their self-efficacy and awareness gradually, which was ended up in noticing the importance of perseverance and patience. As a result, pre-service ECE teachers have sustained their motivation because of the raising of awareness about technology use in education. Pre-service ECE teachers’ statements are as follows:

Before, technology integration in education was not necessary. I see it as a must now. (a self-evaluation statement, from a portfolio)

... production gave me a sense of pleasure, frankly... I think of everything as technology-oriented at the moment. Of course, if the facilities in the school are sufficient, I will definitely use technology, it (the course) has been very useful. (P-6, from interview)

My first view of the course was that I started with bias. I ended this process with curiosity and perseverance. Now, I have an idea about how necessary technology is and how important it is in my future career. (anonymous, from online course evaluation form)

I think that all prejudices against technological applications have been destroyed within the scope of this course thanks to the teacher's attitude. Even if I have difficulties in the first stages, I think this difficulty makes learning more permanent. (anonymous, from online course evaluation form)

The change in motivation and attitudes of pre-service ECE teachers also fostered them to transfer their practical experiences to the field. It was observed that pre-service ECE teachers could address potential problems by using technology in education, make plans, use new applications effectively for pedagogical purposes, and produce new and original pedagogical materials using applications. Also, it was frequently mentioned that taking an active role in the whole process and gaining experience increases the permanence of acquired knowledge and skills.

I started doing things in ten minutes that used to take two hours, and I really want to use in the future most of the apps I used. (P-2, from interview)

While I was a beginner, I think I have mastered many things now. And at the same time, I had the opportunity to send the applications I made during the lesson to my friends' children. I had the opportunity to share the things I produced due to the pandemic. I started using those programs to do something new. In the family education class, I prepared family participation posters and invitation cards... (P-5, from interview)

The change of mentioned psychological factors led the pre-service ECE teachers to reach a level where they can learn on their own. Self-regulated learning about educational technology was often mentioned as “I can find/ search/ learn” in pre-service ECE teachers’ portfolios and project reports. The most important proof of this was the presentations of the final projects, including the materials they have developed with an application that they have not learned before.

I liked every step of the lesson very much. I am sorry that this lesson is over now. You [instructor] helped me a lot, especially with building self-confidence. Now I say that I can learn everything if I want. (anonymous, from online course evaluation form)

Do not avoid learning applications. Knowing is freedom. When you need to do something in an application, the pleasure of doing it yourself without help cannot be described. Time will pass and maybe the applications we use now will get old. Do not neglect to follow the innovations, learn and try. (a suggestion herself in the future, from a final project self-assessment report)

I really had a hard time choosing a suitable outcome for myself and designing a suitable material for the outcome. Although I had difficulty in the selection part, I think that I did not have that much difficulty while designing the materials. (a statement, from a final project self-assessment report)

The last, pre-service ECE teachers showed high collaborative skills about project/material development and the ability for peer mentoring as mentioned in the learning process section. So, it can be said that they can support their peer for curriculum studies, designing activity plans, learning new applications.

## Discussion and implications

This research was aimed at developing the technology integration knowledge and skills of future pre-service ECE teachers. The instructional technologies course was created collectively based on a praxeological approach by the pre-service ECE teachers with the guidance of the instructor. As a result, it was revealed that pre-service teachers were not used to such teaching/learning methods, their content preferences for the course were decided on applications with visual and auditory elements because of the target audience. Also, they generally had a positive attitude towards technology in education, even though they had a negative attitude towards technology. At the end of the course, while enhancing their technology integration competencies, several learning outcomes were accomplished, such as self-regulated learning, collaborative project development, building sustainable motivation, peer mentoring, transferring acquired skills in different contexts. Therefore, it is possible to discuss the study results from two perspectives. The first one is about developing technology integration competencies of pre-service ECE teachers. Secondly, it concerns the contributions of the praxeological approach in terms of teacher education.

### Pre-service ECE teachers’ technology integration skills development

First of all, it was observed in the praxeological designed course that low awareness, attitudes, and skills of pre-service ECE teachers towards instructional technologies were disincentive factors. Similarly, the studies show that attitudes and beliefs are significant factors for technology integration skills (Abbitt, 2011; Seufert et al., 2021; Tezci, 2011; Tondeur et al., 2017; Yerdelen-Damar et al., 2017). Also, observed prior knowledge levels differences of teachers were also a filter to think about the design of the course to subsume all participants. In this regard, one of the course’s aims became both raising awareness and developing a positive attitude towards instructional technologies while benefiting the individual differences. Additionally, participants were encouraged to the enhancement of their self-efficacy, and question effective technology use in ECE. Therefore, it is highly recommended to determine and include teaching/learning strategies about pre-service teachers' attitudes and awareness taking into account their pre-skills into instructional technologies training rather than focusing on only skill development in the training content.

It is emphasized that technology courses for pre-service ECE teachers should be customized with the technologies and applications by covering the purpose of the preschoolers' education (Masoumi, 2021; Xie et al., 2019). Accordingly, the learning process was structured with domain-specific technologies and scaffolding mechanisms (task-based lists and case studies) for content. The education provided takes the approach of introductory to speciality, taking into account the content suggested by the participants. The course was designed gradually to develop pre-service teachers' technology integration skills in ECE; applications utilization for general technology knowledge, learning outcome-oriented digital material design, and the adaptation of technologies to different educational contexts, respectively. This process internalizes the transition from technological knowledge to technological pedagogical content knowledge proposed by Koehler and Mishra (2005). Hence, since the learning process was experience-based, skill-oriented, and supportive to the attitude changing, pre-service ECE teachers were motivated to learn both individually and collectively through activities. Also, Neumann et al. (2021) indicated that detached courses for instructional technologies cannot represent the realities in the classrooms, therefore, practice-based courses should be offered. Current research also has recommended providing more learning opportunities and practical experiences for pre-service teachers in this regard (Howard et al., 2021; Masoumi, 2021; Neumann et al., 2021). It is suggested to design course content in which field-specific examples and practices are provided for the development of pre-service teachers' knowledge and skills in this field and to test their effectiveness. It is also advised to take into account the differences in technological knowledge, subject knowledge, and pedagogy knowledge of pre-service teachers in the design of these activities. For more details, it can be investigated how the pre-service teachers perceive field-specific applications in terms of usefulness, benefit, and easiness, and to determine the factors that affect their preferences in future research.

Considering the learning outcomes at the end of the learning process, it was observed that the pre-service ECE teachers' technology integration skills were improved. The most important changes were that they can predict alternatives, decide necessary material types based on learning outcomes, select appropriate applications and learn by themselves. Hence, at the educational technologies course end, the pre-service ECE teachers' lesson plans and materials became more detailed and they believe that this training is valuable for them in the future (Neumann et al., 2021). In addition, they reached some extra outcomes, such as collaborative studying, self-regulated learning, motivation and perseverance, peer mentoring, and transferring their knowledge. Online break-out room activities mediated computer-supported collaborative learning to reveal these outcomes. Although individual differences affect computer-supported collaborative learning, social sensitivity enables people to get together respectfully, constructively, and cohesively, so that equitable collaborations (Isohätälä et al., 2021). With this understanding, democratic participation was supported as well. Moreover, collaborative activities triggered their motives because of witnessing peers’ facing challenges and overcoming them. So that, the gained sustainable motivation and perseverance can pave the continuous learning of future ECE teachers to follow self-regulated professional development. Therefore, there is a need to investigate how computer-supported collaborative activities (in our case, it was break-out rooms) with peer mentoring leverage productivity and solidarity to attain new skills and knowledge. Furthermore, it is also suggested to examine online collaborative studies' ice breakers to foster active and democratic participation of the pre-service teachers.

### Praxeological learning approach for teacher education

The praxeological-learning approach can make crucial contributions to teacher education in terms of many characteristics, such as taking responsibility, deciding on the purpose and process, respect for diversity; in short, being a democratic individual and a teacher. This study derived that pre-service ECE teachers’ motives made a substantial contribution because of being accustomed to this method over time to overcoming inabilities, hesitations, and prejudices. Furthermore, their perseverance manifested itself after the performance accomplishments, vicarious experiences, and peer support. Thanks to this flexible understanding, pre-service ECE teachers experienced the freedom of decision and the satisfaction of production-based study. Winterbottom and Mazzocco’s (2016) research results about teacher education also indicated that the pre-service teachers believed attendance of an experience of academic praxeological-learning develops pedagogical and social skills development and self-actualization. Hence, it can be inferred that they demonstrated improvement in different areas besides academic qualifications. However, the studies in the educational area focus more on measurable and predetermined questions and restricted results (Biesta, 2007; Pascal & Bertram, 2012; Vandenbroeck et al., 2012). Moreover, this issue narrows down the educational desirable opportunities, which makes the deficit of democratic education is apparent (Biesta, 2007). Therefore, this approach, without pre-determination of the continuum, allows all participants to equally engage in the research and educational paths.

On the other hand, this approach is discussed in specific for instructional technology education in teacher education programs. Although pre-service teachers are expected to have technology skills, contrary to popular belief, it is seen that they might not be enough digitally fluent (Martín et al., 2020). The fact indicates that pre-service teachers during their education need to be involved in a sort of transformation period to be able to have a digitally competent teacher mind map. Inasmuch as, teachers are expected to be able to show some roles such as “learner”, “leader”, “collaborator”, “designer” in digital educational settings (International Society for Technology in Education, 2021). Therefore, the praxeological approach in instructional technology education can provide wider opportunities for pre-service teachers to comprehend the nature of technological pedagogical content knowledge. For this reason, it seems important to uncover some teaching/learning strategies to ease integration of the initial challenges to the learning process within the courses designed with the praxeological-learning method in further research. Consequently, developing pre-service teachers’ digital competencies is a complex and multifaceted process to be needed detailed investigations (Howard et al., 2021; Masoumi, 2021; Tondeur et al., 2012), hence, we believe that the praxeological approach posits alternative ways in this context.

## Conclusion

In conclusion, in this study, the praxeological approach was used in both the research and the teaching methods. Even if initial challenges were released as attitudes, awareness, and competencies, it was observed that pre-service ECE teachers took on their learning responsibilities as the subject of the learning process. Thus, they actively participated in the teaching process and positive changes took place in the attitudes, knowledge, and skills about the use of technology in education. Moreover, it was seen that pre-service teachers can learn on their own, design projects collectively, mentor their peers, and transfer knowledge and skills to different contexts at the end of the semester. It has been observed that they tend to continue these skills hereafter. As a result, the praxeological approach used in instructional technology education in teacher education programs leads to a crucial digital transformation to be ready to become future teachers. This course, including self and group learning activities, is also seen as a preliminary experience of future professional development training. Hopefully, this course will contribute to the construction process of their professional development as digitally competent future teachers.

### Limitations

This study has some limitations both in terms of the implementation process and research methodology. On one hand, a few students did not have a computer during the course time, so that, they completed the semester with their mobile devices. To minimize the inequality of opportunity, offline support was provided and compatible mobile applications were suggested for them. Of course, it is possible to say that this approach was not suitable for a few students who preferred traditional education and considered this course tiring.

On the other hand, the nature of this research requires commitment and a bond between the participants and the instructor, however, this may lead to misrepresentations instead of reflecting actual results in real life (Pascal & Bertram, 2012). Nevertheless, to prevent this, the role of the second researcher stands as a filter during data collection and analysis. Also, democratic participation in the research does not follow the ordinary linear path to study, therefore, it makes it hard to conduct and conceptualize, and makes unpredictable (Vandenbroeck et al., 2012). Hence, we collected different types of data during the research, which assisted in the confirmation and comparison of the findings. The study is not generalizable as it was carried out in a contextualized subject-specific area and limited group, however; we consider it serves as a good example in this field. Considering the current circumstances, we believe that it’s high time to employ a praxeological approach in teacher education to tackle Biesta’s (2007) “democratic deficit” in education.

## Supplementary Information

**Additional file 1.** Learning process of the Instructional Technologies Course for pre-service ECE teachers.

**Additional file 2.** Interview protocol.

## Data Availability

The datasets used and/or analyzed during the current study are available from the corresponding author on reasonable request.

## Abbreviation

ECE: Early childhood education

## References

[CR1] Abbitt JT (2011). An investigation of the relationship between self-efficacy beliefs about technology integration and Technological Pedagogical Content Knowledge (TPACK) among preservice teachers. Journal of Digital Learning in Teacher Education.

[CR2] Biesta G (2007). Why “what works” won’t work: Evidence-based practice and the democratic deficit in educational research. Educational Theory.

[CR3] Bolstad R (2004). The role and potential of ICT in early childhood education: A review of New Zealand and international literature.

[CR4] Braun V, Clarke V, Cooper H (2012). Thematic analysis. Handbook of research methods in psychology: Research designs.

[CR5] Contini, A., Bertolini, C., Manera, L., Martin, I., Schlemmer, D., Kiefer, M., Nousiainen, T., Merjovaara, O., Gözen, G., & Pagano, A. (2018). Guidelines for digital storytelling in early childhood education [Project Report]. https://ec.europa.eu/programmes/erasmus-plus/project-result-content/fede56b0-7a42-4bde-9b4d-463871c653c2/GUIDELINE_English%20language.pdf

[CR6] Creswell JW, Creswell JD (2018). Research design: Qualitative, quantitative, and mixed methods approaches.

[CR7] Dong C, Xu Q (2020). Pre-service early childhood teachers’ attitudes and intentions: Young children’s use of ICT. Journal of Early Childhood Teacher Education.

[CR8] Formosinho J, Oliveira-Formosinho J (2012). Towards a social science of the social: The contribution of praxeological research. European Early Childhood Education Research Journal.

[CR9] Howard SK, Tondeur J, Ma J, Yang J (2021). What to teach? Strategies for developing digital competency in preservice teacher training. Computers & Education.

[CR10] International Society for Technology in Education. (2021). ISTE standards for educators ISTE. https://www.iste.org/standards/iste-standards-for-teachers

[CR11] Isohätälä J, Näykki P, Järvelä S, Bakers MJ, Lund K (2021). Social sensitivity: A manifesto for CSCL research. International Journal of Computer-Supported Collaborative Learning.

[CR12] Jung J, Ottenbreit-Leftwich A (2020). Course-level modeling of preservice teacher learning of technology integration. British Journal of Educational Technology.

[CR13] Kerckaert S, Vanderlinde R, van Braak J (2015). The role of ICT in early childhood education: Scale development and research on ICT use and influencing factors. European Early Childhood Education Research Journal.

[CR14] Koehler MJ, Mishra P (2005). What happens when teachers design educational technology? The development of Technological Pedagogical Content Knowledge. Journal of Educational Computing Research.

[CR15] Lauricella AR, Herdzina J, Robb M (2020). Early childhood educators’ teaching of digital citizenship competencies. Computers & Education.

[CR16] Martín SC, González MC, Peñalvo FJG (2020). Digital competence of early childhood education teachers: Attitude, knowledge and use of ICT. European Journal of Teacher Education.

[CR17] Masoumi D (2021). Situating ICT in early childhood teacher education. Education and Information Technologies.

[CR18] Miles MB, Huberman AM (1994). Qualitative data analysis: An expanded sourcebook.

[CR19] Morfoniou K, Voulgari I, Sfyroera M, Gouscos D (2020). Digital games and the emergence of problem solving processes: A case study with preschool children. International Conference on the Foundations of Digital Games.

[CR20] Neumann KL, Alvarado-Albertorio F, Ramírez-Salgado A (2021). Aligning with practice: Examining the effects of a practice-based educational technology course on preservice teachers’ potential to teach with technology. TechTrends.

[CR21] Öner D (2020). The using technology and digital games in early childhood: An investigation of preschool teachers' opinions. Inonu University Journal of the Graduate School of Education.

[CR22] Pascal C, Bertram T (2012). Praxis, ethics and power: Developing praxeology as a participatory paradigm for early childhood research. European Early Childhood Education Research Journal.

[CR23] Patton MQ (2002). Qualitative research & evaluation methods.

[CR24] Polly D, Byker E (2020). Considering the role of zone of proximal development and constructivism in supporting teachers’ TPACK and effective use of technology. Revista Educación a Distancia.

[CR25] Romero-Tena R, Barragán-Sánchez R, Llorente-Cejudo C, Palacios-Rodríguez A (2020). The challenge of initial training for early childhood teachers. A cross sectional study of their digital competences. Sustainability.

[CR26] Schina D, Esteve-González V, Usart M, Lázaro-Cantabrana J-L, Gisbert M (2020). The integration of sustainable development goals in educational robotics: A teacher education experience. Sustainability.

[CR27] Segal-Drori O, Ben Shabat A (2021). Preschoolers’ views on integration of digital technologies. Journal of Childhood, Education & Society.

[CR28] Seufert S, Guggemos J, Sailer M (2021). Technology-related knowledge, skills, and attitudes of pre- and in-service teachers: The current situation and emerging trends. Computers in Human Behavior.

[CR29] Tezci E (2011). Factors that influence pre-service teachers’ ICT usage in education. European Journal of Teacher Education.

[CR30] Tondeur J, van Braak J, Ertmer PA, Ottenbreit-Leftwich A (2017). Understanding the relationship between teachers’ pedagogical beliefs and technology use in education: A systematic review of qualitative evidence. Educational Technology Research and Development.

[CR31] Tondeur J, van Braak J, Sang G, Voogt J, Fisser P, Ottenbreit-Leftwich A (2012). Preparing pre-service teachers to integrate technology in education: A synthesis of qualitative evidence. Computers & Education.

[CR32] Van Scoter J, Ellis D, Railsback J (2001). Technology in early childhood education: Finding the balance.

[CR33] Vandenbroeck M, Roets G, Roose R (2012). Why the evidence-based paradigm in early childhood education and care is anything but evident. European Early Childhood Education Research Journal.

[CR34] Verbruggen S, Depaepe F, Torbeyns J (2021). Effectiveness of educational technology in early mathematics education: A systematic literature review. International Journal of Child-Computer Interaction.

[CR35] Winterbottom C, Mazzocco PJ (2016). Reconstructing teacher education: A praxeological approach to pre-service teacher education. European Early Childhood Education Research Journal.

[CR36] Xie K, Vongkulluksn VW, Justice LM, Logan JAR (2019). Technology acceptance in context: Preschool teachers’ integration of a technology-based early language and literacy curriculum. Journal of Early Childhood Teacher Education.

[CR37] Yerdelen-Damar S, Boz Y, Aydın-Günbatar S (2017). Mediated effects of technology competencies and experiences on relations among attitudes towards technology use, technology ownership, and self efficacy about Technological Pedagogical Content Knowledge. Journal of Science Education and Technology.

[CR38] Zilka GC (2021). Attitudes of preservice kindergarten teachers toward the integration of computers and the reduction of the digital divide in kindergartens. Educational Technology Research and Development.

[CR39] Zomer, R. N. (2014). *Technology use in early childhood education: A review of the literature* [Unpublished master's thesis]. University of Ontario.

