# Supplementary material for: Development of pre-service early childhood teachers’ technology integrations skills through a praxeological approach
Source: Int J Educ Technol High Educ. 2022 Jul 28;19(1):36. doi: 10.1186/s41239-022-00344-8 (PMC9331027; doi:10.1186/s41239-022-00344-8)
Supplement: Supplementary file 1 — Additional file 1. Learning process of the Instructional Technologies Course for pre-service ECE teachers. [file 41239_2022_344_MOESM1_ESM.docx]

**Additional file 1.** Learning process of the Instructional Technologies Course for pre-service ECE teachers

| Week | Parts | Topic | Content | In-class activity | Out-class activity | Decisions/Outcome for Process |
| --- | --- | --- | --- | --- | --- | --- |
| 1. | Introduction | Introduction |  |  |  | Syllabus preparation agreement |
| 2. |  | Fundamentals of the Instructional Technologies | Basic concepts | Expository teaching, Discussions |  | Determination of the learning method, assessment, and content |
| 3. |  | Cloud Applications | Google Drive | Task-based implementation |  | Determination of the learning method, assessment, and content |
| 4. | Independent Studies | 2B Visual Materials | Canva | Task-based implementation | 2B visual material development |  |
| 5. |  | Presentations | Prezi | Task-based implementation | Presentation design |  |
| 6. |  | Animations | Powtoon | Task-based implementation | Animation development |  |
| 7. |  | Digital educational games | A group of Web 2.0 game apps | Collaborative  task-based implementation | Game development | Topic/content update on the syllabus |
| 8. |  | Digital stories | Storyjumper, Animoto | Task-based implementation | Digital story development | Portfolio/Self-evaluation |
| 9. | Context Orientation Studies | Problems | Authentic problems in technology-based lessons | Collaborative case studies, Discussions | Project development |  |
| 10. |  | Suggestions | Alternative solutions for problems in technology-based lessons | Collaborative case studies, Discussions | Project development |  |
| 11. | Context-based Studies | Project Presentations |  | Volunteer Presentations, Discussions | Project development |  |
| 12. |  | Project Presentations |  | Presentations |  | Final Project |
| 13. |  | Project Presentations |  | Presentations |  |  |
| 14. |  | Project Presentations |  | Presentations |  |  |
