# Supplementary material for: Development of pre-service early childhood teachers’ technology integrations skills through a praxeological approach
Source: Int J Educ Technol High Educ. 2022 Jul 28;19(1):36. doi: 10.1186/s41239-022-00344-8 (PMC9331027; doi:10.1186/s41239-022-00344-8)
Supplement: Supplementary file 2 — Additional file 2. Interview protocol. [file 41239_2022_344_MOESM2_ESM.docx]

**Additional file 2**

**Interview Protocol**

Dear…

We invite you to the research titled “Development of pre-service early childhood teachers’ technology integrations skills through a praxeological approach” conducted by -BLINDED FOR REVIEW PROCESS-. Your participation in this study is entirely voluntary. We are going to interview you about the purpose of the study and we will collect the data during this interview. The data collected from this study will be used for research purposes and your personal information will be kept confidential; however, your data may be used for publication purposes.

If you read and approve this form, it will mean that you agree to participate in the research voluntarily. However, you also have the right not to participate in the study or to stop working at any time after participating.

Participant’s sign

Date

**Semi-structured questions***

1) You took this course as a mandatory requirement. Did you have any expectations from this course? If so, what were they?

2) You contributed to course design as a colleague. What was it like for you to plan the content of your course?

3) The teaching method of the course was carried out mainly based on implementations according to your (class) suggestions. Did this method have an impact on your technology integration skills? If so, could you explain with an example?

4) How did you think the interactions in classwork and project groups during the semester affect your technology competencies? Could you explain with an example?

5) Could you tell me how you felt during this course? How your motivation was affected?

6) At the end of the course, do you think you achieved your goals at the beginning of the semester?

7) Is there anything else you want to mention?

* translated from Turkish
